# Optimal controllers resembling postural sway during upright stance

**DOI:** 10.1371/journal.pone.0285098

**Published:** 2023-05-02

**Authors:** Hedyeh Jafari, Thomas Gustafsson

**Affiliations:** Control Engineering Group, Department of Computer Science, Electrical and Space Engineering, Luleå University of Technology, Luleå, Sweden; National Institute of Technology, India (Institute of National Importance), INDIA

## Abstract

The human postural control system can maintain our balance in an upright stance. A simplified control model that can mimic the mechanisms of this complex system and adapt to the changes due to aging and injuries is a significant problem that can be used in clinical applications. While the Intermittent Proportional Derivative (IPD) is commonly used as a postural sway model in the upright stance, it does not consider the predictability and adaptability behavior of the human postural control system and the physical limitations of the human musculoskeletal system. In this article, we studied the methods based on optimization algorithms that can mimic the performance of the postural sway controller in the upright stance. First, we compared three optimal methods (Model Predictive Control (MPC), COP-Based Controller (COP-BC) and Momentum-Based Controller (MBC)) in simulation by considering a feedback structure of the dynamic of the skeletal body as a double link inverted pendulum while taking into account sensory noise and neurological time delay. Second, we evaluated the validity of these methods by the postural sway data of ten subjects in quiet stance trials. The results revealed that the optimal methods could mimic the postural sway with higher accuracy and less energy consumption in the joints compared to the IPD method. Among optimal approaches, COP-BC and MPC show promising results to mimic the human postural sway. The choice of controller weights and parameters is a trade-off between the consumption of energy in the joints and the prediction accuracy. Therefore, the capability and (dis)advantage of each method reviewed in this article can navigate the usage of each controller in different applications of postural sway, from clinical assessments to robotic applications.

## Introduction

Maintaining balance involves the cooperation of three systems in the human body in a feedback loop: the musculoskeletal system, the sensory system, and the Central Nervous System (CNS). The CNS as the main controller of the balancing loop collects information from the sensory organs in the body such as vision, vestibular, and proprioception. Integrating and processing this information generates the proper motor commands to the musculoskeletal system [1, 2]. Aging or injuries can decline the performance of the human balance system. Therefore, studying human balance motor control is crucial from three perspectives. First, it can develop an understanding and diagnosis of balance impairment conditions in health care such as Parkinson’s disease. Second, it can lead to the design of new assistive devices for rehabilitation to aid individuals in regaining their mobility. Last, it can advance biped or humanoid robots in maintaining stability and movement tasks in complex environments [3].

Many studies have been done to understand the human balance structure and its complex subsystem to capture its complexity and represent simplified models. Generally, the skeletal system is modeled in the sagittal plane as a single link inverted pendulum rotating around the ankle joint [4–7] or double link inverted pendulum rotating around the ankle and hip joints [8, 9]. The multisensory system in an upright stance is the integration of three primary sensors, vision, proprioception, and vestibular. There are few studies about qualitative modeling of this system [10]. However, many studies consider the integration of data provided by these organs as inputs to the CNS affected by neurological time delay and perturbations [11, 12]. Several studies implemented the Extended Kalman Filter (EKF) for estimating the delayed time and compensating the perturbations (measurement noise from sensors) [13, 14].

Despite all efforts to understand the mechanism of the neuromuscular system to maintain balance in an upright stance, the complexity of CNS has made the model for postural control controversial. Many researchers have tried to find a simplified control model to simulate the performance of the postural controller in maintaining the human balance in an upright stance [2, 10, 15–18]. Generally, the postural controller is considered as a Proportional-Derivative (PD) or Proportional-Integral-Derivative (PID) controller that provides joints torque for the skeletal system in a feedback loop [1, 5, 16, 19–22]. Despite the simplicity of this representation, it is an unsophisticated presentation of the postural control, which does not consider the predictability and adaptability of the CNS to the internal and environmental changes. For example, in the presence of the biological transmission time delay, selecting PD’s parameters in a stable region is quite a challenge [23]. To solve this issue, modeling the neural controller in postural sway leads to different methodologies that are more robust to the parameter changes.

Intermittent Proportional Derivative (IPD) controller is utilized in many studies [24–28] in order to improve the robustness issue of the PD controller in the balance feedback loop. The paradigm is based on the idea that the feedback controller can be switched on close to the stable manifold and switched off near an unstable manifold in the phase plane. In other words, instead of equilibrium configuration in the classical feedback PD controller, the stable manifold is the goal that leads to bounded stability [26]. The method is based on an active intermittent control torque applied to the ankle joint and a passive stiffness control to the hip joint. In [29], the author has extended the IPD model by applying independent parameter gains for both the ankle and the hip joint that can explain the mixed ankle-hip strategy. While IPD is more robust to the time delay than the PD controller, its adaptability and predictability to parameter changes are not widely understood.

Another potential approach to mimic the neuromuscular controller is using optimization techniques. This approach’s main advantage is considering the body’s limitations and constraints (e.g., joint torque and angle limit) in formulating the postural sway model. The ability to predict the future is another benefit of this approach that leads to more accurate results. There are increasing number of studies that used optimization techniques [30–38]. In general, the postural controller is formulated as an optimization problem to minimize the postural sway. Other cost functions have been studied, minimizing energy consumption in joints or tracking the body’s momentum. However, to what degree they can mimic the postural sway in an upright stance in case of time delay and parameter changes and their validity to replicate experimental data is a question that needs to be answered.

Validation and justification of the hypothetical simplified postural controllers with the experimental human data is yet an open research direction. In a few articles, the postural controller is defined by system identification techniques based on the experimental data [39–43]. Data-driven controllers are practical approaches to identify the relationship between the inputs to the neural controller and the generated outputs from that in the balancing loop and can be a tool to develop the parameters of the model-based approaches [44, 45]. However, the main drawback is that they can not explicitly describe the dynamics and pathophysiology behind the neuromuscular controller. On the other hand, validating and adjusting the simplified postural control model with human data is a technique that can contribute to a reliable understanding of the postural control model.

This paper aims to evaluate the optimal controllers that can resemble the postural control in an upright stance. Our goal is to find a structure that can mimic the human balance strategy and can be implemented in balance applications. Therefore, we have studied the human-likeliness of three commonly used formulations of the optimal controller (Model Predictive Control (MPC), COP-Based Controller (COP-BC) and Momentum-Based Controller (MBC)) in the literature. Specifically, we evaluated these methods in a human balance feedback structure loop while considering the sensory system’s noise and transmittal time delays. First, in simulation studies, we compared the robustness to different perturbations, the consumption of energy, and the postural strategy of each technique to reveal the human-likeliness of each method. Second, we validated and compared the efficiency of these methods in predicting postural sway through multiple human experimental data in upright stance trials with changes in the vision and proprioception sensory input. Finally, we examined the choice of parameters in the performance of the methods mentioned above and compared the results with IPD controllers. We believe our findings aid the postural sway analysis used in clinical and assistive robotic applications.

## Methods

### Human data collection

The experimental data is part of a larger project collected in the Human Health and Performance Lab at Luleå the University of Technology, Luleå, Sweden [43, 46, 47]. Written informed consent was obtained from all individual participants included in the study. The study design was approved by the Regional Ethical Review Board in Umeå, Sweden (ref no. 2015-182-31) and was organized according to the 1964 Helsinki declaration.

Ten subjects were chosen from a larger study [46] where community-dwelling elderly who have a critical balance posture were selected with a mean age of 75.2(±4.5) years. The Center Of Pressure (COP) data was collected by a force plate with a sampling frequency of 3000*Hz*, while the subjects were asked to stand still and look straight at the fixed point. In order to measure the total time delay subjects did a reaction time test where they had to press the bottom when they received the visual and audio signals as fast as possible. The experiment was repeated for five trials. The angular position of the joints was collected by a Qualisys Motion Capture System with eight cameras and a 200 *Hz* sampling rate. The experiment to measure reaction time is explained in detail in [47].

### Human balance control loop

**Fig 1:**
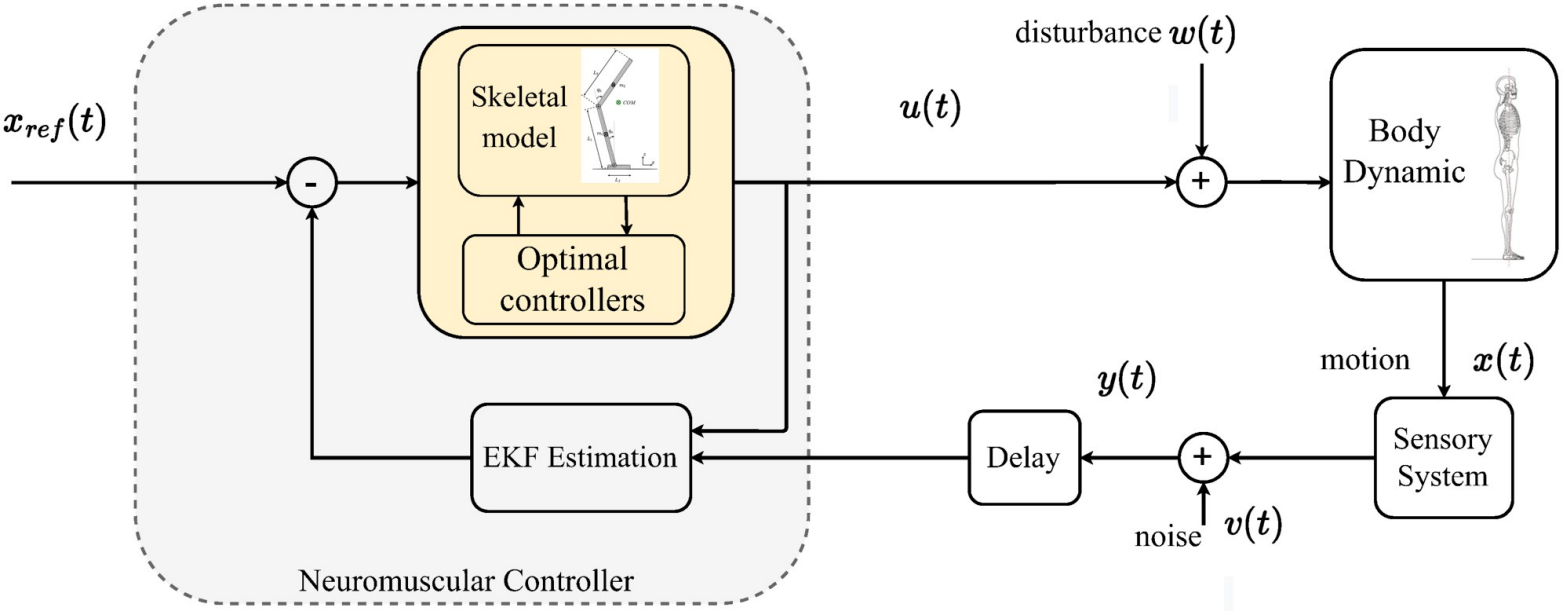
The illustration of the postural controller feedback loop is studied in this paper. The body’s dynamical system is activated by control input *u*(*t*) and affected by disturbances *w*(*t*). The sensory system receives the motion dynamic *x*(*t*) and transfers it to neuromuscular contorted by noise *v*(*t*) and time delay *t*_*d*_. The delayed sensory information and a buffer of the control input up to the current time *u*(*t*) are used in the neuromuscular by EKF method to estimate delayed states. The optimal controller block refers to all optimal methods explained in this work. The reference angular position *x*_*ref*_(*t*) = 0 is considered zero degrees in an upright stance.

Fig 1 presents the block diagram of the closed-loop control structure to stabilize the human body in a quiet stance by the hip and ankle joints.

Body dynamics described in (2) is activated by control input ***u***(*k*) = [*τ*_*a*_, *τ*_*h*_]^⊤^ and exposed to unknown disturbances ***ψ***_*k*_. The states of the system are $x\left(k\right)={\left[q_{a},q_{h},\dot{q_{a}},\dot{q_{h}}\right]}^{⊤}$ which can be derived by solving the nonlinear discrete form at time instance $k\in Z^{+}$ as:

$$\begin{array}{c} x_{k+1}=F\left(x_{k},u_{k}\right)+\psi_{k}, \end{array}$$ (1a)

$$\begin{array}{c} y_{k}=S\left(x_{k}\right)+v_{k}, \end{array}$$ (1b)

where $\psi_{k}\in R^{n_{s}}$ is the applied disturbance to the system. $F:R^{n_{s}}\times R^{n_{u}}\to R^{n_{s}}$ is a nonlinear function describing the dynamics of the skeletal system. $S:R^{n_{s}}\to R^{n_{m}}$ is the vector of measured states affected by noise in neural sensing $v_{k}\in R^{n_{m}}$ and $y\left(k\right)={\left[q_{a}^{m},q_{h}^{m},{\dot{q_{a}}}^{m},{\dot{q_{h}}}^{m}\right]}^{⊤}$ is the vector of measured outputs by sensors. The number of states, inputs and measurements are shown by *n*_*s*_, *n*_*u*_, *n*_*m*_ respectively.

### Skeletal modeling and body dynamics

**Fig 2:**
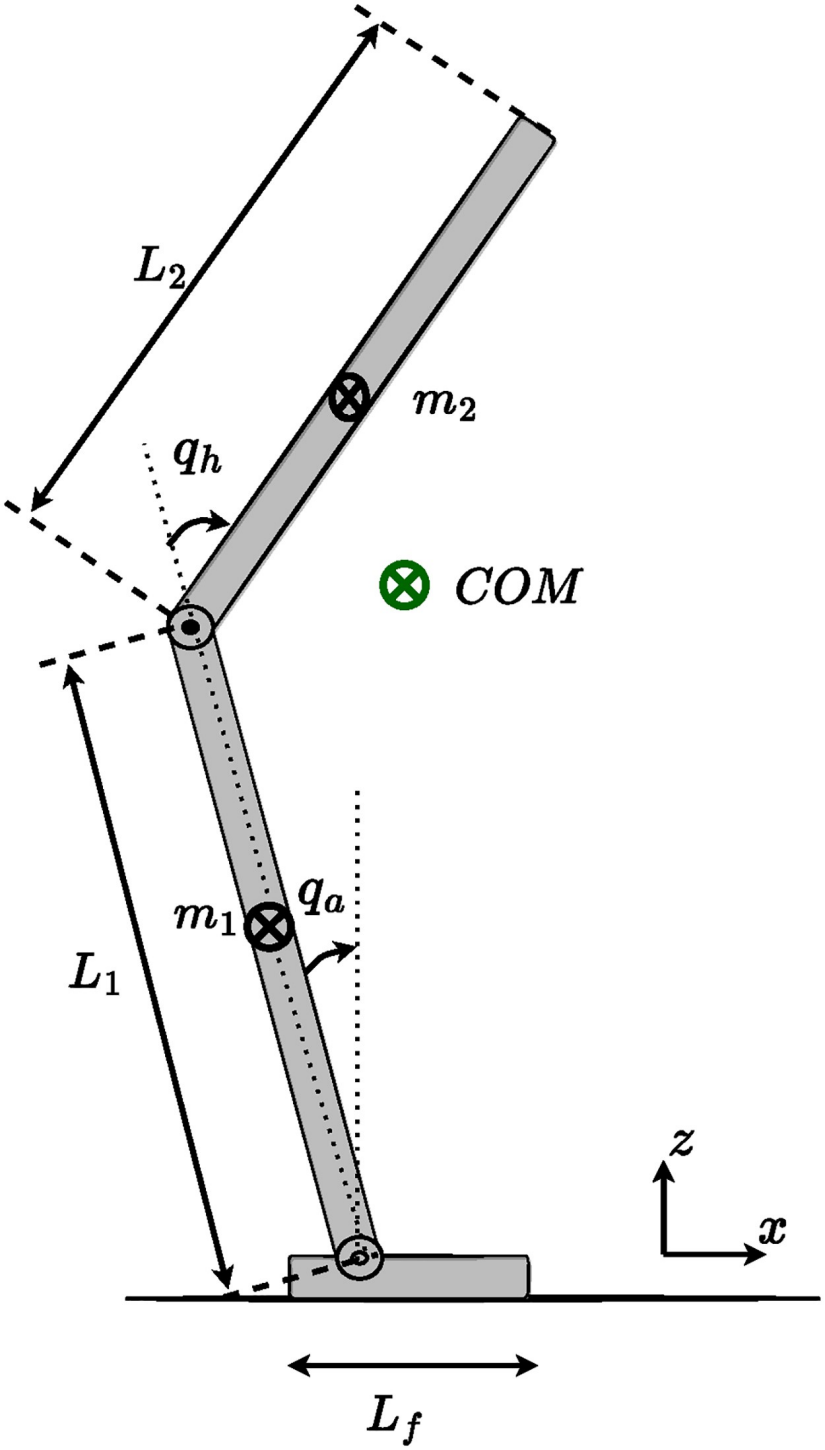
Illustration of two joints dynamical model of the human body in standing position. *q*_*a*_ and *q*_*h*_ represent the angular position of the ankle and hip joints respectively. *COM* is the location of the center of the system’s total mass, while *m*_1_ and *m*_2_ are the mass of the lower body and upper body respectively. *L*_*f*_ is the length of the foot.

The human body in quiet standing can be modeled as a two-link inverted pendulum rotating around the ankle and hip joints, as illustrated in Fig 2 [48]. In this representation, it is assumed that for small perturbations, the knee joint in the lower body and body segments from the hip joint to the head are neglected. However, for more significant perturbation, the human body bends the knee and takes a step to increase the base of support, which this scenario is beyond the scope of this article.

The equation of motion can be written in the following form:

$$\begin{array}{c} M\ddot{q}+C\left(q,\dot{q}\right)+F\left(q\right)=H \end{array}$$ (2)

where ***q*** = [*q*_*a*_, *q*_*h*_]^⊤^ is the angular rotation around ankle (*q*_*a*_) and hip (*q*_*h*_), $M=[\begin{array}{cc} M_{11} & M_{12}\\ M_{21} & M_{22} \end{array}]$ represents the mass matrix with:

$$\begin{array}{cc} M_{11} & =\frac{1}{4}L_{1}^{2}m_{1}+L_{1}^{2}m_{2}+\frac{1}{4}L_{2}^{2}m_{2}\\ & +L_{1}L_{2}m_{2}\;\text{cos}(q_{h})+I_{1}+I_{2} \end{array}$$ (3a)

$$\begin{array}{c} M_{12}=\frac{1}{4}L_{2}^{2}m_{2}+\frac{1}{2}L_{1}L_{2}m_{2}\;\text{cos}\left(q_{h}\right)+I_{2} \end{array}$$ (3b)

$$\begin{array}{c} M_{21}=M_{12} \end{array}$$ (3c)

$$\begin{array}{c} M_{22}=\frac{1}{4}L_{2}^{2}m_{2}+I_{2}, \end{array}$$ (3d)

where *L*_1_ and *m*_1_ represent the length and mass of the lower body, while *L*_2_ and *m*_2_ show the length and mass of the torso, respectively. *I*_1_ and *I*_2_ represent the inertia around the ankle and the hip joint, respectively. It is assumed the center of mass of each segment is in the middle of the segment. $C(q,\dot{q})$ is the vector of Coriolis and Centrifugal terms with:

$$\begin{array}{c} C=[\begin{array}{c} -\frac{1}{2}L_{1}L_{2}m_{2}\;\text{sin}\left(q_{h}\right){\dot{q}}_{h}^{2}-L_{1}L_{2}m_{2}{\dot{q}}_{a}{\dot{q}}_{h}\;\text{sin}\left(q_{h}\right)\\ \frac{1}{2}L_{1}{\dot{q}}_{a}^{2}\;\text{sin}\left(q_{h}\right) \end{array}]. \end{array}$$ (4)

The vector with gravitational force ***F***(***q***) is depicted as:

$$\begin{array}{c} F=[\begin{array}{c} F_{1}\\ -\frac{1}{2}L_{2}m_{2}g\;\text{sin}\left(q_{a}+q_{h}\right) \end{array}] \end{array}$$ (5)

where $F_{1}=\frac{1}{2}g\left(-L_{2}m_{2}\;\text{sin}\;\left(q_{a}+q_{h}\right)-L_{1}m_{1}\;\text{sin}\left(q_{a}\right)+2L_{1}m_{2}\;\text{sin}\left(q_{a}\right)\right)$.

***H*** represents the applied torques vector as:

$$\begin{array}{c} H=[\begin{array}{c} \tau_{a}\\ \tau_{h} \end{array}] \end{array}$$ (6)

where *τ*_*a*_, *τ*_*h*_ are the generated torque at the ankle and hip joint respectively.

### Neural time delays and noise in the sensory system

Balancing requires different sensory systems such as proprioception, vestibular, and vision [5]. Due to the different lengths of the neural path, there are time latencies in transmitting information to the CNS. Besides, less delays exist in processing at the CNS and transducing the information to the musculoskeletal system by applying the executed forces and torques on muscles and joints. In general, the total time is called the neural time delay, and for the body, sway is in the range of ∼ 100 − 300 (*ms*). However, the primary time delays happen in observation and perception of sensory data ∼ 200 − 300 (*ms*) [49]. Aging and impaired functions in any part of the balance control loop can increase the neural time delay, resulting in difficulty of balance and falls [50].

Therefore, the neuromuscular controller receives sensory information as:

$$\begin{array}{c} y_{k-k_{d}}=S\left(x_{k-k_{d}}\right)+v_{k-k_{d}} \end{array}$$ (7)

where *k*_*d*_ is the total measurement delay from sensors, called observation delay.

The neuromuscular controller has to reduce the noise from the received delayed measurement and predict and estimate the current states of the system for further prediction of control actions. Besides, it is assumed that the Neuromuscular controller has a memory of the previous control actions $\left\{u_{k-k_{d}},\ldots ,u_{k-1}\right\}$.

Here, we have used EKF to resemble the prediction and estimation of noisy delayed sensory measurements. The algorithm works in two steps. First, the EKF generates the estimate of the delayed states ${\hat{x}}_{k-k_{d}|k-k_{d}}$ by finding the Kalman gain $G_{k-k_{d}}$ and updating the states error covariance $P_{k-k_{d}|k-k_{d}}$. Next, the states and the state error covariance matrix will be predicted for the next sampling time. The procedure is repeated until it reaches the estimate of the current time step ${\hat{x}}_{k}$ which is explained in more detail in [51].

### Neuromuscular controllers

#### Model predictive controller

The MPC solves an optimization problem at each sampling time for a finite prediction horizon *N* to find the sequence of optimal control inputs. The cost function to be minimized is introduced as:

$$\begin{array}{c} \underset{u}{\text{min}}\;J=\sum_{j=0}^{N-1}\|{\hat{x}}_{k}-x_{r}{\|}_{\Gamma }^{2}+{\left\|u_{k}-u_{r}\right\|}_{\Lambda }^{2}, \end{array}$$ (8)

where $N\in N^{\geq 2}$ is the prediction horizon, ${\hat{x}}_{k}$ is the estimated states vector, ***x***_*r*_ presents the reference states, ***u***_*k*_ and ***u***_*r*_ are the controller action and desired control action, respectfully. The first term, weighted by $\Gamma \in R^{4}$, minimizes the error between the current states and the desired one. The second term, weighted by $\Lambda \in R^{2}$, tracks the error between the produced control action with the desired one.

The cost function (8) is minimized subject to the following constraints:

1. The dynamic of the body in discrete form as in Eq (1).
2. The joint torques (*τ*_*a*_, *τ*_*h*_) are bounded:
  $$\begin{array}{cc} \tau_{a}^{min}<\tau_{a}\left(j\right)<\tau_{a}^{max} & \forall j\in \{0,1,\ldots ,N-1\} \end{array}$$ (9a)
  $$\begin{array}{cc} \tau_{h}^{min}<\tau_{h}\left(j\right)<\tau_{h}^{max} & \forall j\in \{0,1,\ldots ,N-1\} \end{array}$$ (9b)

The MPC solves the mentioned above optimal control problem for a finite horizon *N* at each sampling time. The optimum results for the predicted horizon are u* = {*u*(0)_*opt*_, *u*(1)_*opt*_, …, *u*(*N* − 1)_*opt*_}. Then the first optimal value *u*(0)_*opt*_ is applied to the body to obtain the angular position and velocity.

#### Center of pressure based controller

The structure of this controller is similar to the MPC controller with the difference in using COP in the objective function. Theoretically the COP is calculated from the torque applied at the toe *τ*_*t*_ and the vertical component of ground reaction force *f*_*v*_ applied on the foot [49]:

$$\begin{array}{c} COP=\frac{\tau_{t}}{f_{v}}. \end{array}$$ (10)

The control torque at the toe and the ground reaction are defined from the Euler-Lagrange equations from the potential and kinetic energy as described in [49]: The cost function can be written as follows:

$$\begin{array}{c} \underset{u}{\text{min}}\;J=\sum_{j=0}^{N-1}\|x_{COP}-x_{COP}^{d}{\|}_{\alpha }^{2}+{\left\|u_{k}-u_{r}\right\|}_{\beta }^{2}, \end{array}$$ (11)

where $x_{COP}^{d}$ is the desired distance for ground pressure to keep the balance which is zero in this case. α∈R and $\beta \in R^{2}$ are the weights of error of COP and control action respectively. In addition to the constraints in MPC, the horizontal position of COP should always remain in the base of support, which in quiet standing with two joints is equivalent to the length of the foot *L*_*f*_:

$$\begin{array}{c} L_{f}^{min}<x_{COP}\left(j\right)<L_{f}^{max}\;\forall j\in \left\{0,1,\ldots ,N-1\right\} \end{array}$$ (12)

#### Momentum based controller

MBC is widely used in controlling the humanoid robots [52–54]. In [55], the usage of this controller on the mediolateral balance is studied. Here, we implemented this controller based on [34]. The main goal is to find the joint torques by the whole body momentum. The whole body momentum *h* can be derived as follows:

$$\begin{array}{c} h=A_{G}\dot{q}, \end{array}$$ (13)

where $\dot{q}={\left[{\dot{q}}_{a},{\dot{q}}_{h}\right]}^{⊤}$ is the vector of angular velocities and *A*_*G*_ is the centoridal momentum matrix calculated based on the description in [56]. The momentum rate change can be calculated by taking the derivative of Eq 13 and through the contact forces by the Newton-Euler equations.

$$\begin{array}{c} \dot{h}=A_{G}\ddot{q}+{\dot{A}}_{G}\dot{q}=[\begin{array}{cc} 1 & -x_{COM}\\ 0 & 1 \end{array}][\begin{array}{c} \lambda_{1}\\ \lambda_{2} \end{array}]+[\begin{array}{c} 0\\ -Mg \end{array}], \end{array}$$ (14)

$$\begin{array}{c} \lambda =[\begin{array}{c} x_{COP}f_{cy}\\ f_{cy} \end{array}], \end{array}$$ (15)

where λ is the contact wrench and *f*_*cy*_ is the contact force applied to the foot in the vertical axis.

The MBC optimization can be written in the following form by minimizing the momentum rate and angular acceleration error:

$$\begin{array}{c} \underset{\ddot{q},\lambda }{\text{min}}\;J=\sum_{j=0}^{N-1}\|\dot{h}-{\dot{h}}^{d}{\|}_{\Phi }^{2}+{\left\|\ddot{q}-{\ddot{q}}^{d}\right\|}_{\Omega }^{2} \end{array}$$ (16)

$$\begin{array}{c} s.t.Equation\;14 \end{array}$$ (17)

$$\begin{array}{c} {\ddot{q}}_{min}<\ddot{q}<{\ddot{q}}_{max} \end{array}$$ (18)

$$\begin{array}{c} L_{f}^{min}<x_{COP}<L_{f}^{max} \end{array}$$ (19)

where ${\dot{h}}^{d}$ is the desired momentum rate, ${\ddot{q}}^{d}$ is the reference angular acceleration, Φ and Ω are the weight in the minimization term of the momentum rate change and the angular acceleration, respectively. The weights are chosen according to to [34] to guarantee stability. By finding the optimal value for $\ddot{q}$ and λ, the control torque *τ* can be computed by solving the inverse dynamics.

#### Intermittent PD controller

In this method the generated torque for each joint *τ* = [*τ*_*a*_, *τ*_*h*_] from the neuromuscular controller consists of three parts:

$$\begin{array}{c} \tau =\tau^{b}+\tau^{s}+\tau^{i} \end{array}$$ (20)

where *τ*^*b*^ is the bias torque apply to compensate for the gravity force:

$$\begin{array}{c} \tau^{b}=-g[\begin{array}{c} \left(\frac{m_{1}L_{1}}{2}+m_{2}L_{1}\right)\;\text{sin}\;q_{a}^{ref}+\frac{m_{2}L_{2}}{2}\;\text{sin}\;q_{a}^{ref}+q_{h}^{ref}\\ \frac{m_{2}L_{2}}{2}\;\text{sin}\;q_{a}^{ref}+q_{h}^{ref} \end{array}] \end{array}$$

*τ*^*s*^ is the torque related to the stiffness and damping in the muscles as follows:

$$\begin{array}{c} \tau^{s}=-[\begin{array}{c} K_{a}\left(q_{a}-q_{a}^{ref}\right)+B_{a}{\dot{q}}_{a}\\ K_{h}\left(q_{h}-q_{h}^{ref}\right)+B_{h}{\dot{q}}_{h} \end{array}] \end{array}$$

with *K*_*a*_, *K*_*h*_, *B*_*a*_, *B*_*h*_ represent the stiffness and damping parameters of ankle and hip joints respectively. The last term *τ*^*i*^ in the Eq 20 shows the intermittent control torque. Here, we implemented this torque as in [25] contrary to other literature that considers the intermittent control on a single inverted pendulum around the ankle, which has considered two degrees of freedom around the ankle and hip. In their representation, the Virtual Inverted Pendulum (VIP) is considered to calculate the rotation around the ankle by a virtual link to the whole body Center Of Mass (COM) as:

$$\begin{array}{c} q_{COM}=tan^{-1}(\frac{x_{com}}{y_{com}}), \end{array}$$ (21)

where $x_{com}=m_{1}\frac{L_{1}}{2}\;\text{sin}\;q_{a}+m_{2}\left(L_{1}+\frac{L_{2}}{2}\right)\;\text{sin}\left(q_{a}+q_{h}\right)$ and $y_{com}=m_{1}\frac{L_{1}}{2}\;\text{cos}\;q_{a}+m_{2}\left(L_{1}+\frac{L_{2}}{2}\right)\;\text{cos}\;\left(q_{a}+q_{h}\right)$. Then the error between the estimated value of the angular location of COM by EKF and the reference value ($q_{com}^{er}=q_{com}^{ref}-{\hat{q}}_{com}$) is used to generate the control action only on the ankle joint as follows:

$$\begin{array}{c} \tau^{i}=[\begin{array}{c} C\\ 0 \end{array}], \end{array}$$

$$\begin{array}{c} \text{with}\{\begin{array}{cc} C=Pq_{com}^{er}+D{\dot{q}}_{com}^{er} & \text{if}\;q_{com}^{er}\left({\dot{q}}_{com}^{er}-\alpha q_{com}^{er}\right)>0\\ C=0 & \text{otherwise}\; \end{array} \end{array}$$ (22)

where *P*, *D* are the proportional and derivative parameters, respectively, and *α* is the switching parameter. To have a wider stable region based on the value of *P* and *D*, we chose *α* = 0.4 as discussed in [23], and the controller becomes active in the first and third quadrants of the phase plane.

## Results

### Simulation results

The human body parameters used for the simulation are summarized in Table 1. The simulation results were run on MATLAB and ‘ode23t’ function was used to solve the numerical integration. For an accurate comparison, noise perturbation is simulated by a white noise signal with a standard deviation of 0.005 for all the trails. The neural delay of 200(*ms*) is chosen based on the literature [49]. The EKF is run with a time step the same as the sampling time and fixed covariance matrices of measurement noise and disturbances are chosen empirically as *Q* = [*diag*(0.01, 0.01, 0.01, 0.01)] and *R* = [*diag*(0.1, 0.1, 0.01, 0.01)].

**Table 1:** Body characteristics.

	Mass	Length	Inertia
Foot	*m*_0_ = 3.71 *kg*	*L*_0_ = 0.158 *m*	*I*_0_ = 0.025 *kg*.*m*^2^
Shank	*m*_1_ = 11.41 *kg*	*L*_1_ = 0.78 *m*	*I*_1_ = 0.35 *kg*.*m*^2^
Torso	*m*_2_ = 50.14 *kg*	*L*_2_ = 0.73 *m*	*I*_2_ = 0.25 *kg*.*m*^2^

The stiffness and damping parameter in the IPD controller are chosen as *K*_*a*_ = 500 (*Nm*/*rad*), *K*_*h*_ = 380 (*Nm*/*rad*), *B*_*a*_ = 30 (*Nms*/*rad*), *B*_*h*_ = 30 (*Nms*/*rad*) according the measurements and equations provided in [24, 57]. The optimization is done by MATLAB’s ‘fmincon’ function. The objective function gains in the simulation are chosen empirically so that the outcomes remain stable and sway in the base of the support region. The prediction horizon of *N* = 10 with a nominal sampling time of 0.01 *s* was assumed for the prediction. Table 2 summarized the bounded parameters used in the simulation of optimal controllers methods. The bounds are approximated based on [31, 58].

**Table 2:** Parameters bounds and limitations.

	*L*_*f*_	*τ*_*a*_	*τ*_*h*_	*q*_*a*_	*q*_*h*_
Min	-0.08 *m*	-20 *N*.*m*	-40 *N*.*m*	-0.35 *rad*	-0.53 *rad*
Max	0.22 *m*	20 *N*.*m*	40 *N*.*m*	0.53 *rad*	0.87 *rad*

**Fig 3:**
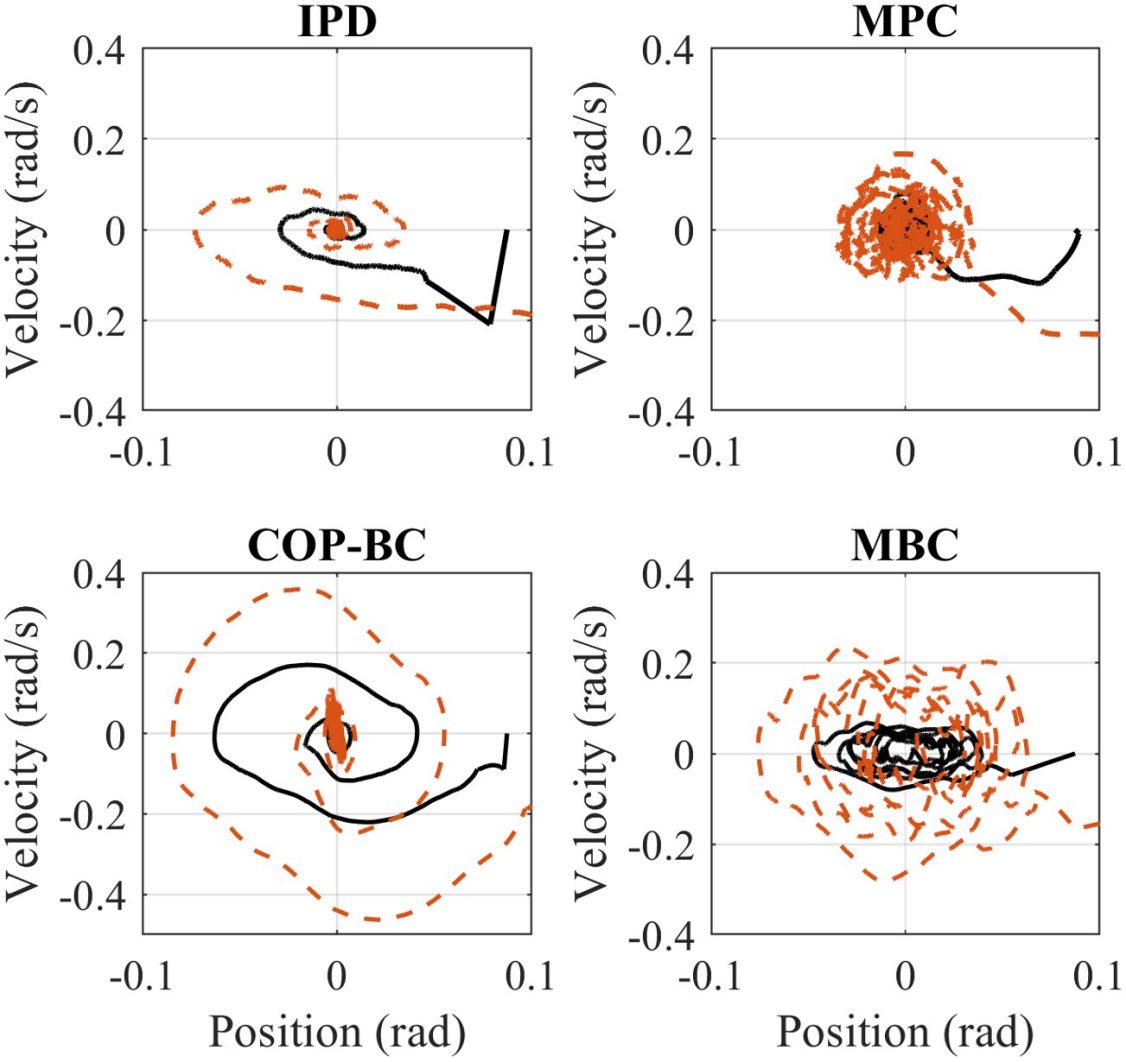
Phase portraits comparison of the mentioned methods for the ankle joint. The solid black line indicates the small perturbation and the dashed red line illustrates the higher perturbation.

**Fig 4:**
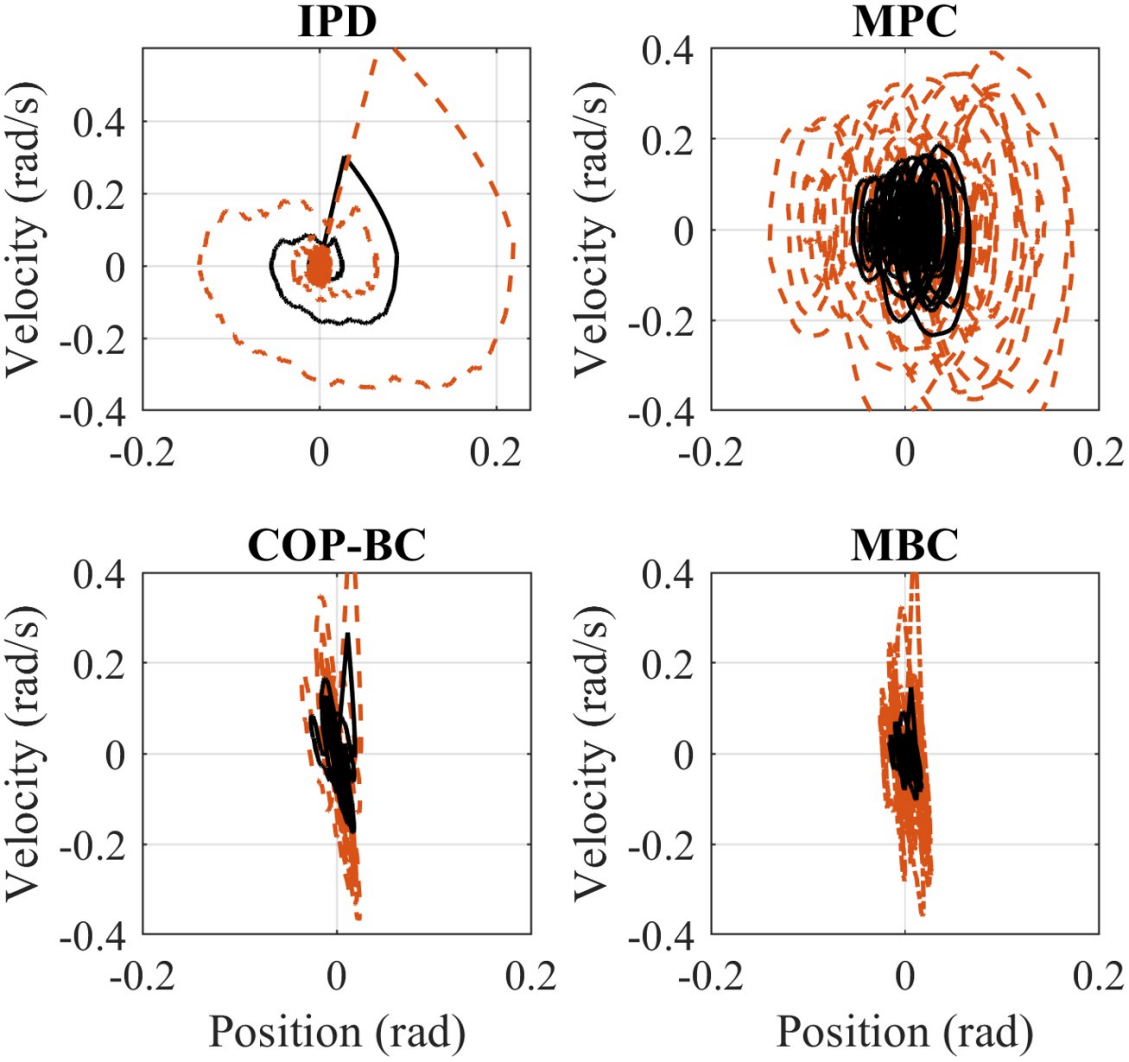
Phase portraits comparison of the mentioned methods for the hip joint. The solid black line indicates the small perturbation and the dashed red line illustrates the higher perturbation.

We simulated each controller starting at two different initial states to compare the performance to perturbations such as external force applied to the body. We assumed an initial state of *q* = [0.0873 0]^*T*^ (*rad*) for a small perturbation initial state of *q* = [0.2618 0]^*T*^ (*rad*) as a higher perturbation. Figs 3 and 4 shows the simulation results with the best-tuned parameter for each method for both ankle and hip joints, respectively. The body stabilizes around zero in all methods for both small and higher perturbations. However, it can be seen that IPD andCOP-BC controllers converge slightly faster to the stable position, while on the other hand, MBC and MPC show more oscillation around zero in ankle and hip joint respectively. Expectedly, increasing the perturbation results in higher amplitude in both angular position and angular velocity in all methods. This analysis is important, especially in the case of push recovery in robotic applications such as humanoids. Considering fast convergence in push recovery and the ability to handle constraints, COP-BC controller can outperform other controllers. It should be highlighted that the results are displayed based on the best-tuned parameter. The choice of parameters can generally affect the controller outcomes’ convergence and settling time. More investigation on the controller parameters is discussed in the discussion section.

**Fig 5:**
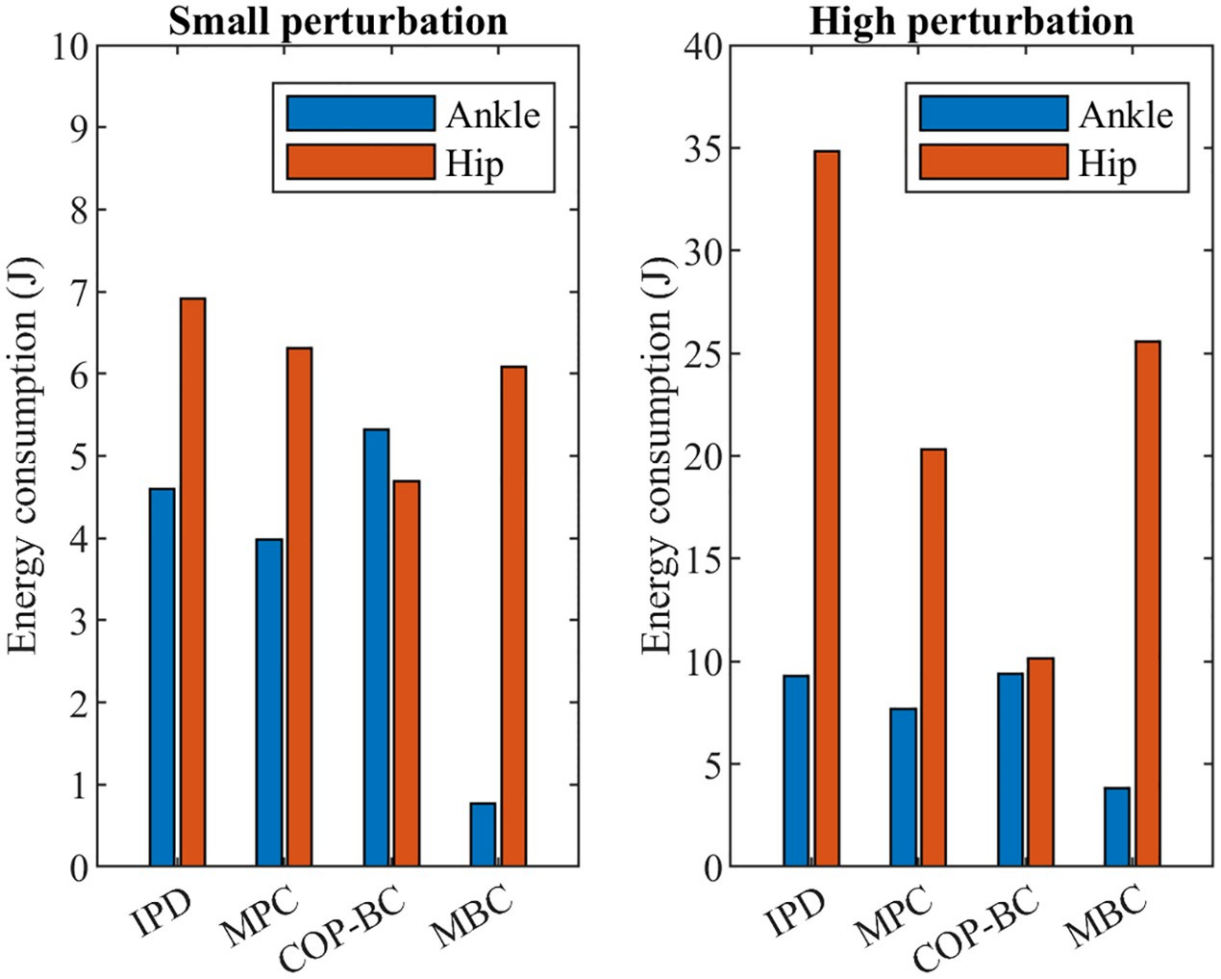
Energy consumption at each joint for different methods.

Other vital factors for comparing the controller are the strategy of balance and the energy consumption in joints. The joint energy is calculated from joint torque *τ* and angular velocity $\dot{q}$ as $E=\int \tau \dot{q}dt$. Fig 5 compares the energy consumed in each joint by each method for different perturbed sway. It can be seen that IPD controller uses more energy at each joint than optimal methods in both small and high perturbation. It is also clear that with higher perturbed sway, the efforts to maintain balance in all the joints will increase and with higher sway, the hip joint consumes more energy than the ankle joint. In the optimal controller methods MBC controller uses significantly lower energy at the ankle compared to the hip joint even with smaller sway, indicating higher efforts of the upper body to stabilize the body in this method. COP-BC controller consumes almost equal energy in both joints to maintain balance in both small and high perturbations.

**Fig 6:**
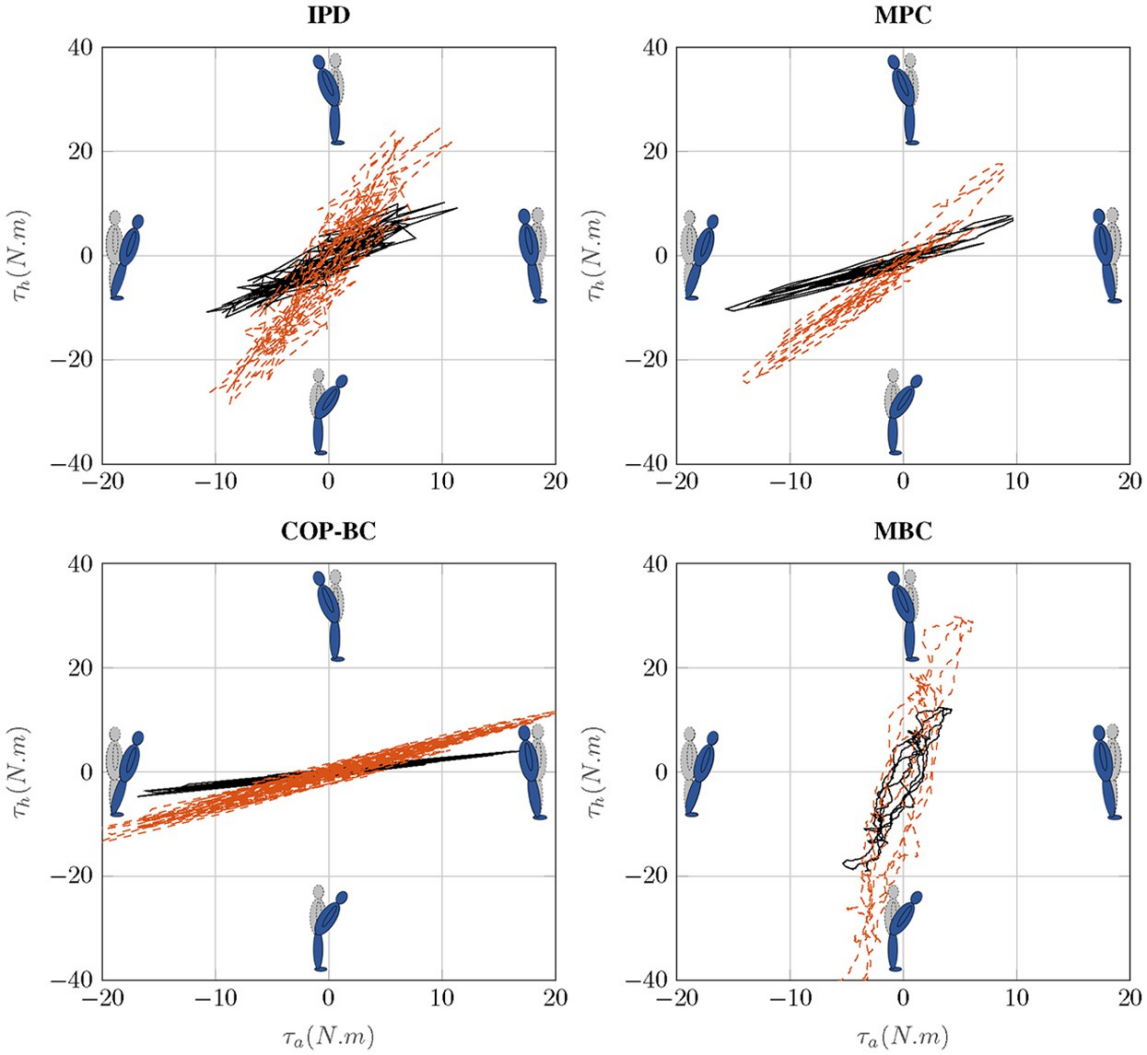
Hip versus ankle joints torque for different methods. The solid black line indicates the small perturbation and the red dashed line indicates the higher perturbation.

In [59] shows that the changes in control strategy from ankle to hip strategy can be seen by changes in joints torque trajectories. [58] mentions that in hip strategy, the ankle plantar flexion torque is smaller than the hip. Considering these studies, each method’s value of joint torque trajectories is plotted in Fig 6. While, as expected, higher perturbation cause shifting from ankle to hip strategy to maintain balance, it is apparent that for small sway IPD and MPC use both ankle and hip joint (ankle-hip strategy). Surprisingly, MBC mainly utilizes a hip strategy to stabilize the body. Although this result confirms the assumption in [60] that in MBC controllers in robotic applications only the hip is actuated to keep the balance, it does not simulate the human postural sway behavior to maintain balance since the human data shows ankle and ankle-hip strategy even in perturbed sway [61]. COP-BC controller mainly uses ankle strategy which shows the robustness of this method to the perturbed postural sway.

### Experimental results

To compare the controllers with the human data, first, we used the measured angular position of the joints as reference data for all the controllers to obtain the cost function gains. An optimization problem was solved to find the gains of the controller as

$$\begin{array}{c} \underset{q,w}{\text{min}}\;\sum_{j=0}^{T}{\left\|q_{human}-q\right\|}^{2}, \end{array}$$ (23)

where *T* is the total time samples (30 *s*) and ***q*** is the vector of the angular position of each joint. Table 3 summarizes the obtained gains for each controller.

**Table 3:** Controllers gains.

MPC	Γ = [5000 ± 1900, 8000 ± 1000, 7000 ± 1000, 8500 ± 1000] Λ = [0.02 ± 0.002, 0.01 ± 0.001]
COP-BC	*α* = 12 ± 2, *β* = [0.9 ± 0.1, 0.8 ± 0.2]
MBC	Φ = [83 ± 2, 85 ± 2], Ω = [1500 ± 300, 1300 ± 200]
IPD	P = 0.7 ± 0.2, D = 352 ± 40

**Fig 7:**
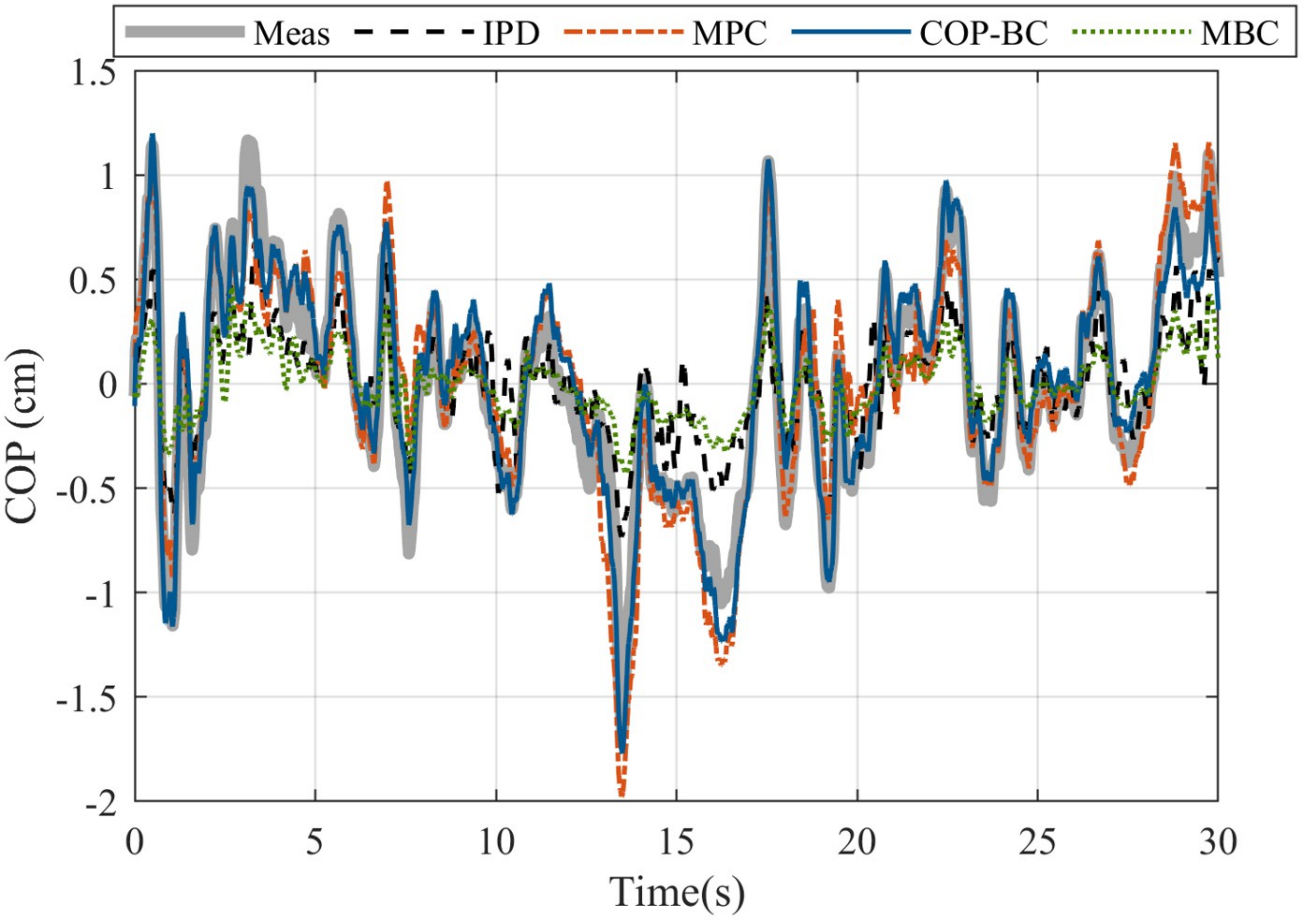
One step ahead prediction of COP with different methods. The subject body parameters are *M* = 67 *kg*, *L* = 1.68 *m*. The noise is estimated as white noise with a standard deviation of 0.005. The reaction time of the subject is 0.310 *s*.

**Fig 8:**
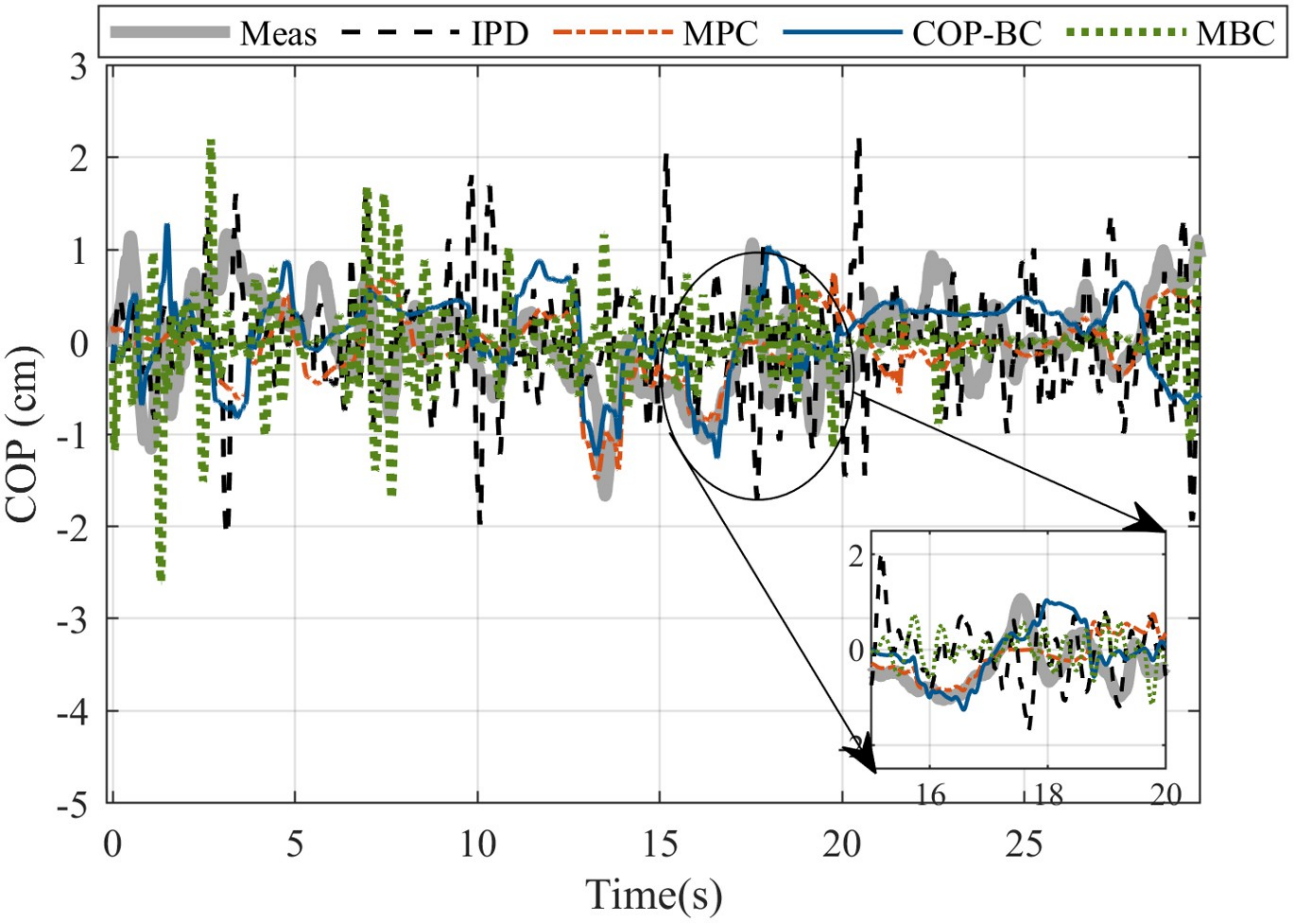
COP validation of measured experimental data of a random subject in the data set with the result of the generated COP of each method for the total time of the prediction. The subject body parameters are *M* = 67 *kg*, *L* = 1.68 *m*. The noise is estimated as white noise with a standard deviation of 0.005. The reaction time of the subject is 0.310 *s*.

Later on, to have a fair resemblance, we simulated all controllers with the initial point of human data measurement, the related body characteristics, and the obtained gains. Fig 7 shows the result for the COP prediction of a random subject in the data set for one step ahead prediction where the human measurement is known at each sampling time as well as Fig 8 that shows the result for prediction for the total measured time of (30 *s*) where only the initial point is known from the human data.

The results are validated by three criteria: Root Mean Square Error (RMSE) and Variance accounted for (VAF) and Power Spectral Density (PSD) in the frequency domain. VAF represents how much the estimated output is similar to the measured data. A higher percentage shows more similarity.

$$\begin{array}{c} VAF=(1-\frac{var(COP_{meas}-COP_{est})}{var(COP_{meas})})*100 \end{array}$$ (24)

Table 4 presents the RMSE and VAF calculated for both prediction for a step ahead and prediction for the total duration of the experiment. According to the table, optimal approaches generally have more accurate predictions than the IPD approach. While theCOP-BC approach has the slightest error and more accurate prediction than other methods. It should be highlighted that while the VAF metric provides a rough approximation of the accuracy of a prediction, it is not a valid indicator for assessing one-step-ahead prediction. This is because the VAF primarily measures the ability of the controller to track the measured COP, whereas the objective is to validate the controllers’ ability to stabilize the position and track the zero angular position.

**Table 4:** Mean± standard deviation of RMSE and VAF for the prediction of COP of ten subjects with different methods.

	One-step-ahead		Total duration of experiment	
	RMSE(*cm*)	VAF%	RMSE(*cm*)	VAF%
MPC	0.1 ± 0.07	96 ± 5	0.6 ± 0.1	53 ± 4
COP-BC	0.1 ± 0.04	96 ± 2	0.5 ± 0.05	45 ± 6
MBC	0.25 ± 0.1	77 ± 3	0.7 ± 0.04	19 ± 3
IPD	0.43 ± 0.15	56 ± 8	0.8 ± 0.07	12 ± 4

**Fig 9:**
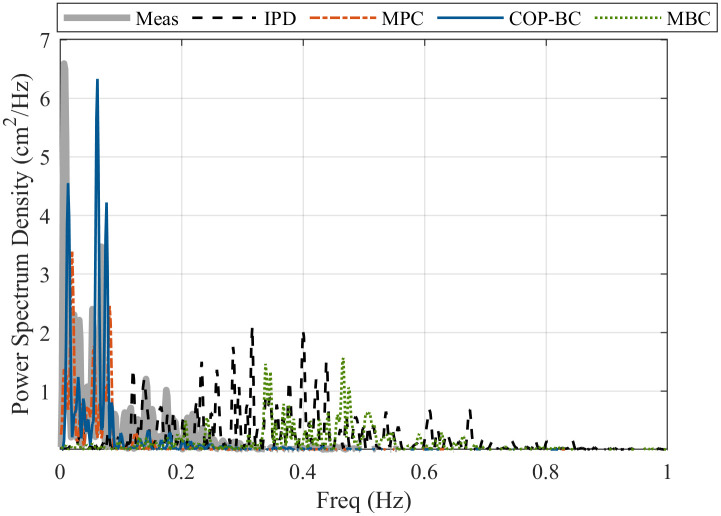
PSD comparison of measured experimental data of a random subject in the data set and the mentioned methods.

However, since approximating individual noise and disturbance may not be accurate, validation in the frequency domain is more reliable. PSD is a common method to analyse the COP in studying postural sway. Here, we used the Lomb-Scargle function in MatLab to calculate the PSD that is preferable in case of short time series to detect low frequencies. Fig 9 compares the PSD calculated for the COP obtained with different methods compared with measured COP of a random subject. It can be seen that only MPC andCOP-BC approaches have similar PSD in small frequencies (below 0.1 *Hz*), compared to the human data.

## Discussion

The choice of weights and feedback parameters in the controller can affect the performance of the controllers. Here, we studied the robustness of each controller to the parameter changes as well as the energy consumption in the joints. For the IPD controller and the optimal controller (MPC andCOP-BC), we changed the parameters so that the controller’s results stay stable and in the range of body joints movements. However, as described in the methodology section, the weight in MBC is chosen as the null space projection of *A*_*G*_ matrix and the stability is sensitive to this weight. Therefore, there is limited freedom to change this weight. Although this can be a limitation, further works need to be done to investigate the stable region of this controller.

**Fig 10:**
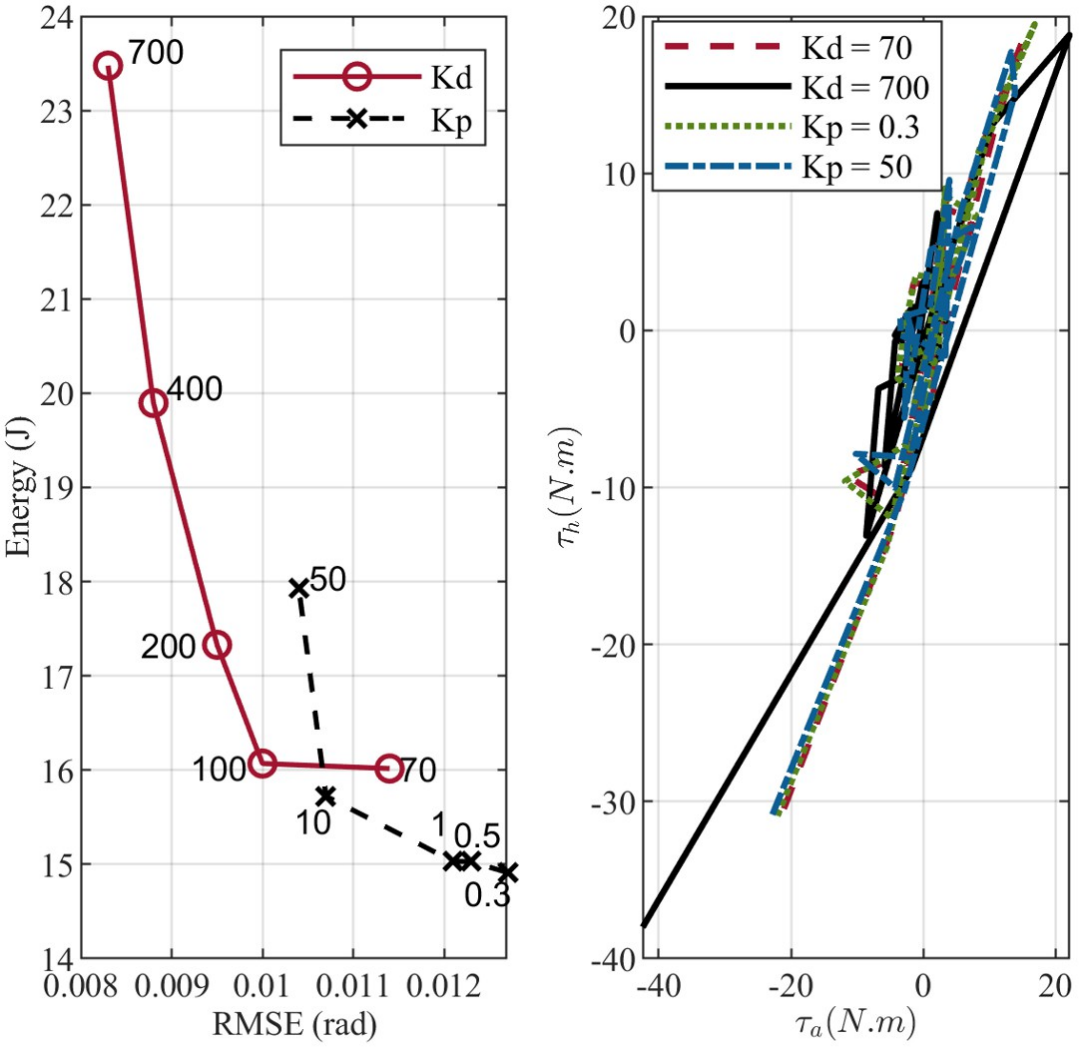
Evolution of changing controllers gain in IPD controller. The upper plot shows the effect of gain change on the total energy consumption in the joints and the RMSE. The lower plot represents the effect on the joints’ torques and standing strategy.

**Fig 11:**
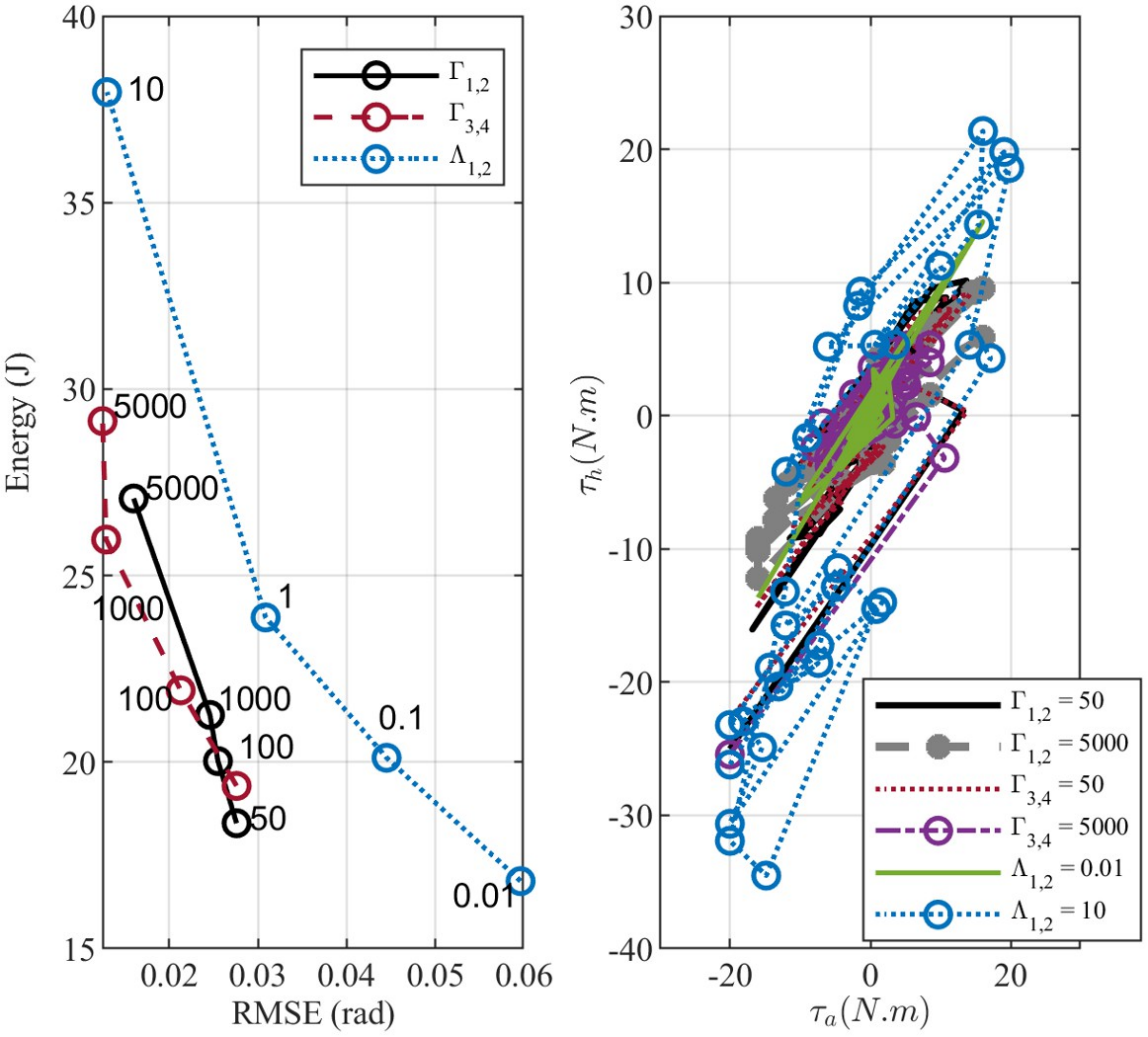
Evolution of changing weights in the optimization of the MPC controller. The upper plot shows the effect of gain change on the total energy consumption in the joints and the RMSE. The lower plot represents the effect on the joints’ torques and standing strategy.

**Fig 12:**
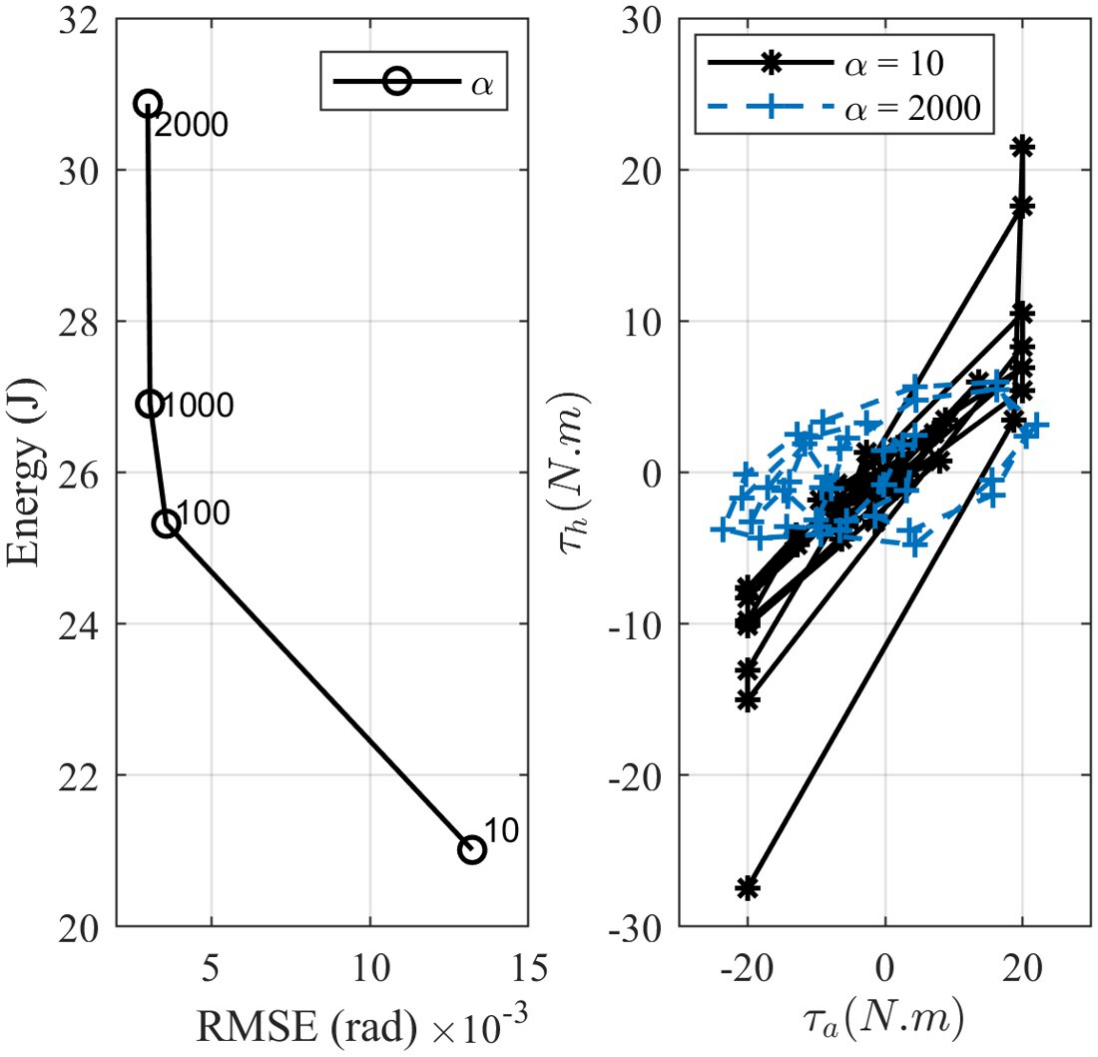
Evolution of changing the COP distance error’s weight (*α*) inCOP-BC controller. The upper plot shows the effect of gain change on the total energy consumption in the joints and the RMSE. The lower plot represents the effect on the joints’ torques and standing strategy.

Fig 10 study the effect of changing the proportional *K*_*p*_ and derivative *K*_*d*_ gain in IPD controller. The energy versus error plot shows that the error between the angular and reference positions decreases as the controller gain increases. At the same time, ankle and hip joints need more effort to maintain balance. Interestingly, the same trend can be observed by changing weights in the objective function for both MPC andCOP-BC controller methods as shown in Figs 11 and 12. This indicates that the change of the controller parameters in the stable region is a trade-off between the energy consumption in the joints and the accuracy of the prediction. This is an essential factor to consider in designing robotic applications. Finding the optimal parameter based on energy consumption and accuracy is in the form of the Pareto front optimization, which is deferred to our future work.

Moreover, we investigated whether parameter changes will influence the balance strategy. As shown in Fig 10 IPD controller uses an ankle-hip strategy despite changes in the controller gains. This is almost the same case for the MPC controller (Fig 11). However, increasing the gain of the error reduction term in theCOP-BC objective function results in favoring the ankle strategy to stabilize the body (Fig 12). When designing robot applications, robustness to parameter changes may be desirable. Therefore, IPD and MPC controllers are more attractive. On the other hand, in the clinical application of the postural sway model, the relation between parameters or weights and postural strategy is an essential factor. Since, by aging or postural related disease, the human body uses more hip, hip-ankle strategy rather than ankle strategy to maintain balance [62, 63]. The results about theCOP-BC, confirm the finding in the [63] where they found the COP information valuable and plausible to generate postural sway. They also found COP metrics sensitive to the parameter changes of the active controller, where the COP obtained by their method can show the difference between younger and older adults’ balance control. Besides, the presence of tactile and proprioception sensors under the feet [64] makes consideration of COP information in the postural sway controllers essential in posturography and clinical applications.

We compared the three most common optimization methods that can mimic postural sway in quiet standing with IPD controller. We chose IPD controller since it solves the stability issue of the PD controller specifically in case of time delays [24]. However, future work should consider other methodologies, such as delayed PD or Proportional-Derivative Acceleration (PDA) controller [65–67], to compare the optimal controllers. PDa feedback controller, where control action is switched on and off based on the sensory dead zone, is a potential methodology to consider specifically in case of larger time delays [68–70]. Furthermore, the intermittency here is based on activating the PD controller based on the state-dependent mechanism. However, other intermittent controller paradigms introduced in [71, 72] and the human likeliness of each paradigm can be studied as future work.

In this work, we utilize EKF to estimate the neural time delay and the sensory noise. However, in spite of the simplicity of EKF in predicting the time delays and reducing the measurement noise, further estimation methods can be studied. Finally, on a broader level, research should also determine the three joints model without locking the knee to study the impact of time delays on the control strategies and utilizing the stepping strategy to maintain balance in a quiet stance.

## Conclusion

In this paper, we investigated optimal control structures mimicking the postural sway by considering the physical body dynamic as two links inverted pendulum and comparing it with the widely used IPD controller. In addition to benefiting from using joint and body constraints, our results show that optimal control techniques consume less energy in the joints and have a more accurate prediction of the control input to the joints with higher accuracy compared to the IPD controller. However, the straightforward implementation and information about the muscles’ damping and stiffness is still an advantage of the IPD method in clinical applications.

In optimal techniques, our results revealed that although the MBC can somewhat predict the control output to the joints, the balance strategy differs from what the human body uses, especially for significant perturbation. Besides, the choice of weight to maintain the controller in a stable region needs more effort than other approaches. Improving the performance of this controller to be more similar to human behavior in postural sway; future work is required to study the additional term to the cost function (Eq 16) that consider the changes of COP.

Both MPC andCOP-BC optimal controllers showed promising results in both simulation and experimental comparison. However, COP-BC controller uses less energy in the hip and mainly uses ankle strategy in postural sway, which is preferable in robotic applications.

We also observed that tuning the parameter is a trade-off between the consumed energy in the joints and the accuracy of the prediction. These findings suggest that, while IPD controller is not superior to other methods, it benefits from simplicity and can be used in clinical and robotic applications. While for more accuracy and less energy consumption, one can benefit from optimal controllers.

Although our finding can be a helpful aid in choosing the balance controller structure for the balance applications, it has some limitations. First, to carefully compare the human likeliness of the different approaches, it is desirable to consider the stepping strategy in higher perturbation. Therefore, future research should study the capacity of different methodologies with a three-link inverted pendulum considering the knee joint. Second, more efforts are needed to investigate the methods considering the model-based structure of the muscular system in the human balance loop.
